# Distinctive features of the oropharyngeal microbiome in Inuit of Nunavik and correlations of mild to moderate bronchial obstruction with dysbiosis

**DOI:** 10.1038/s41598-023-43821-4

**Published:** 2023-10-03

**Authors:** Mathilde Flahaut, Philippe Leprohon, Nguyen Phuong Pham, Hélène Gingras, Jean Bourbeau, Barbara Papadopoulou, François Maltais, Marc Ouellette

**Affiliations:** 1 Centre de Recherche en Infectiologie and Département de Microbiologie, Infectiologie et Immunologie, Faculté de Médecine, Université Laval, Québec City, QC Canada; 2 Department of Medicine, Division of Respiratory Medicine, McGill University Health Center, Montréal, QC Canada; 3 Groupe de Recherche en Santé Respiratoire, Centre de Recherche de L’Institut Universitaire de Cardiologie et de Pneumologie de Québec, Faculté de Médecine, Université Laval, Québec City, QC Canada

**Keywords:** Metagenomics, Microbiome

## Abstract

Inuit of Nunavik are coping with living conditions that can influence respiratory health. Our objective was to investigate associations between respiratory health in Inuit communities and their airway microbiome. Oropharyngeal samples were collected during the Qanuilirpitaa? 2017 Inuit Health Survey and subjected to metagenomic analyses. Participants were assigned to a bronchial obstruction group or a control group based on their clinical history and their pulmonary function, as monitored by spirometry. The Inuit microbiota composition was found to be distinct from other studied populations. Within the Inuit microbiota, differences in diversity measures tend to distinguish the two groups. Bacterial taxa found to be more abundant in the control group included candidate probiotic strains, while those enriched in the bronchial obstruction group included opportunistic pathogens. Crossing taxa affiliation method and machine learning consolidated our finding of distinct core microbiomes between the two groups. More microbial metabolic pathways were enriched in the control participants and these were often involved in vitamin and anti-inflammatory metabolism, while a link could be established between the enriched pathways in the disease group and inflammation. Overall, our results suggest a link between microbial abundance, interactions and metabolic activities and respiratory health in the Inuit population.

## Introduction

Respiratory diseases are associated with considerable socio-economic costs worldwide^1^. Lung cancer, chronic obstructive pulmonary diseases (COPD) and lower respiratory tract infections (LRTI) are all included within the first seven causes of mortality in Canada^1^, with tobacco smoking as the first risk factor. The COVID-19 pandemic has further increased the burden of respiratory diseases. The toll of respiratory diseases is higher in the Inuit of Nunavik (northern Quebec, Canada) compared to the general population^2–4^. The Nunavik Inuit Health Survey in 2004 reported that Inuit are disproportionally affected by LRTI, including tuberculosis^4^. Severe infections early in life can negatively impact the long-term respiratory health. While asthma rates seem lower in Inuit children compared to the Canadian average^5^, the prevalence of COPD or asthma has not been documented in Nunavik adults. To offset this gap in knowledge, a broad health survey that included a comprehensive respiratory health analysis of the Inuit aged 16 and over was recently conducted in Nunavik^6^. In addition to a detailed questionnaire, this survey was further enhanced by oropharyngeal (OP) swabs, as well as spirometry measurements, a lung function test detecting airway obstruction^7–9^. While close to 60% of Nunavimmiut reported one of the symptoms of respiratory health (chronic cough, chronic sputum, wheezing), the vast majority had no airway obstruction as determined by spirometry with 9% showing mild obstruction (GOLD 1) and 5% a moderate obstruction (GOLD 2)^6^. Smoking, and other factors including previous respiratory infection, house crowding, or food security can impact the onset and progression of airway obstruction^6,10,11^.

Increasing evidence gathered in the last decade suggest that the airway microbiome differs between healthy individuals and those with bronchial obstruction^12–14^. Indeed, the airway microbiome composition could protect or exacerbate airway inflammation, lung immunity, or susceptibility to respiratory infections^12,14,15^. While there is variation between studies, Proteobacteria, notably the genus *Haemophilus*, are more abundant in the lung microbiota of COPD patients. On the other hand, Firmicutes, notably the genus *Veillonella*, and Bacteroidetes, notably the genus *Prevotella*, are prevalent in healthy lungs^12,14,16–18^.

While one report has been published on the gut microbiome of Inuit^19^ none has been done for the respiratory microbiome of this Northern population. Inuit have increased rates of respiratory infections at a young age^3^ and have three times higher smoking rates than the average Canadian population^20^. The presence of these risk factors for the development of bronchial obstruction prompted us, in the context of a large survey of respiratory health in Nunavik, to scrutinize the respiratory microbiome. Here, we studied the OP microbiome using whole metagenome sequencing as a proxy of lung microbiome. Indeed, microaspiration of upper airway secretions seems to be the main source of the lung microbiota and the lower airway microbiome is broadly similar to that of the OP airway albeit with a lower biomass within the lung^13,21–23^. Compared to the 16S rRNA gene sequencing, metagenome sequencing allows a more in-depth taxonomic characterization at the species level and provide further functional insights into the human microbiome. Here, we show that the Quebec Inuit population has a distinct respiratory microbiota from other population, and we found several bacterial species associated with either respiratory health or mild-to-moderate bronchial obstruction.

## Results

### Samples and abundance of taxonomic groups

**Figure 1:**
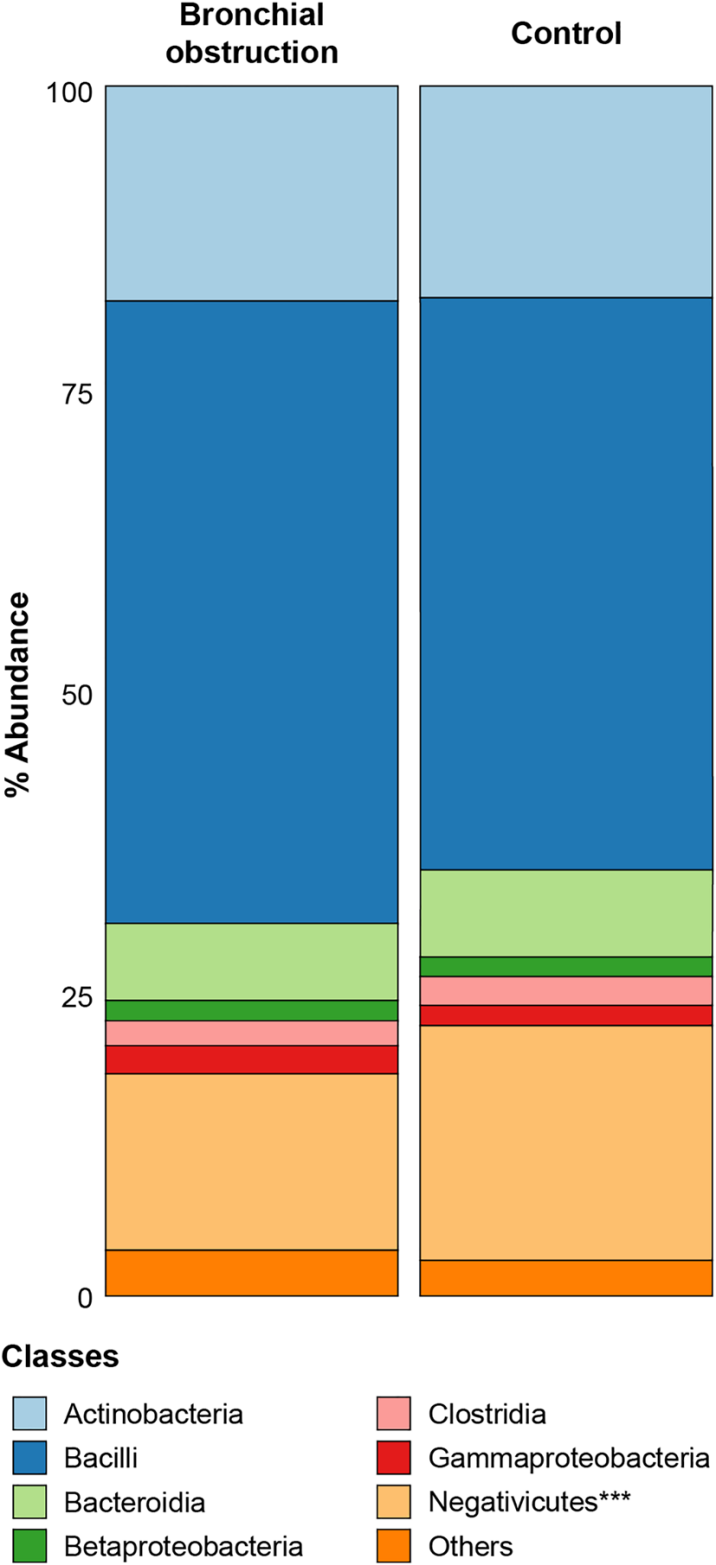
Relative abundance of bacterial classes identified with MetaPhlAn3 in the bronchial obstruction and control groups. Classes on the left are in bronchial obstruction group and classes on the right are in the control group. Only the bacterial classes with a relative abundance higher than 1% are depicted. The abundances for the 25 remaining bacterial classes were summed and shown by the ‘Others’ label. ***, *p-value* < 0.001 with Student’s t-test.

**Figure 2:**
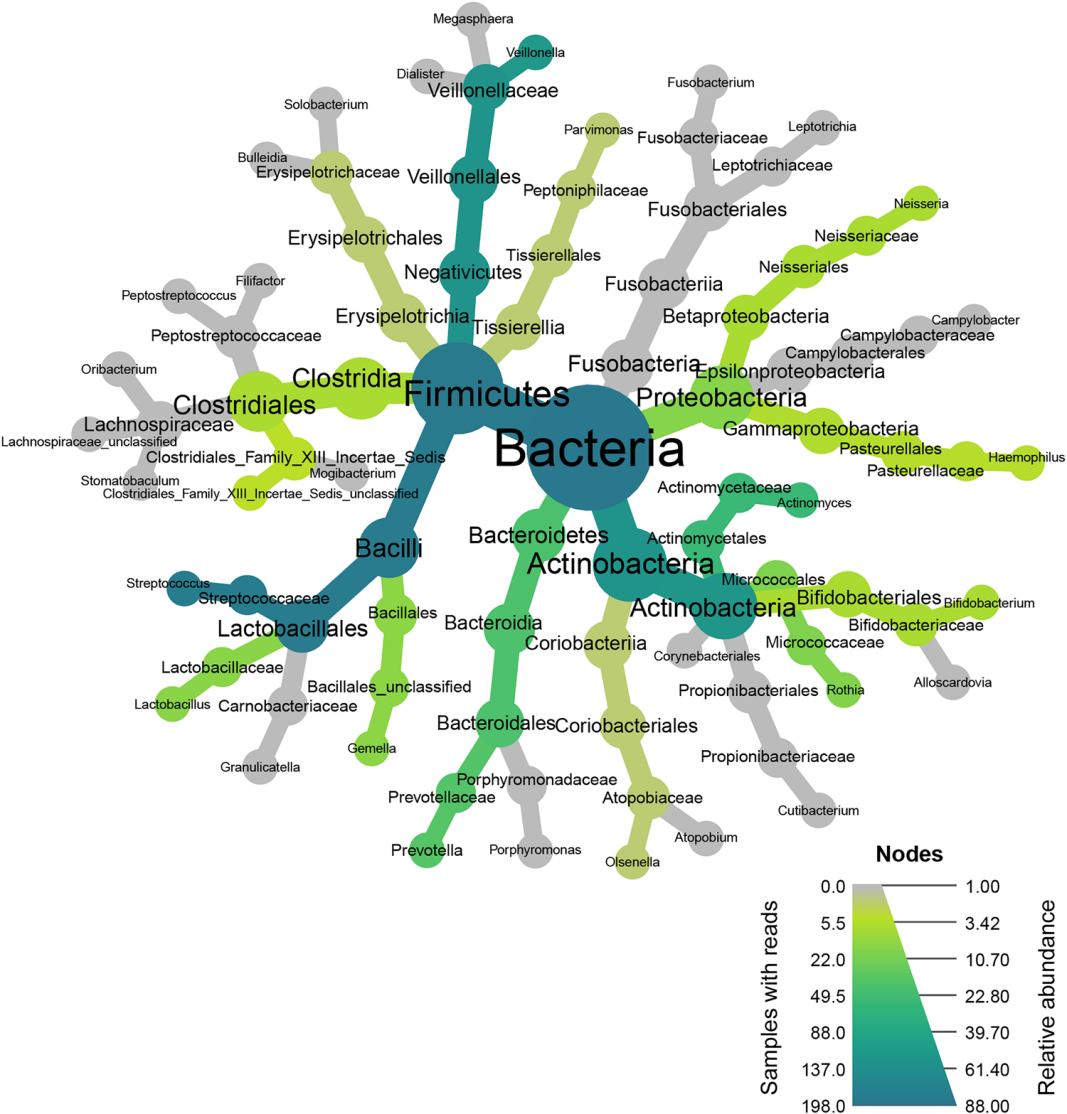
Heat tree for the prevalence and abundance of taxa in the group of 198 participants. The whole group includes 105 participant’s samples with normal spirometry and 93 with evidence of bronchial obstruction. The color code depicts the mean relative abundance of taxa within samples. The prevalence of taxa within the samples group is represented by the size of the nodes.

Out of the 1110 Inuit participants in the Qanuilirpitaa? 2017 survey for respiratory health, 125 participants had a forced expiratory volume in one second (FEV_1_) and forced vital capacity (FVC) ratio below 0.7 that was used to define bronchial obstruction^24^. More than 800 participants had a FEV_1_/FVC ratio above 0.7. We selected 93 participants that met the spirometric criteria for bronchial obstruction. They were matched for age, sex, and geographical location with 105 participants with normal spirometry which was defined as a FEV_1_/FVC ratio > 0.7 and a FVC > 80% predicted (Table S1). The majority (84%) of participants in both groups were smokers. The 198 OP swabs were processed to extract the metagenomes, which were sequenced on a NovaSeq 6000 platform. An average of 104 millions paired-ends reads was obtained per sample. This allowed the detection of 879 taxonomic groups among samples, twenty-six of which were shared by all samples. The most abundant phyla in both the control and mild-to-moderate bronchial obstruction groups were the Firmicutes (70%), the Actinobacteria (18%), the Bacteroidetes (7%) and the Proteobacteria (4%). The three main classes were the Bacilli and Negativicutes, both belonging to the Firmicutes phylum, and the Actinobacteria part of the Actinobacteria phylum (Fig. 1). A total of 509 bacterial species were detected among the 148 bacterial genera detected in the samples (Fig. S1 and Table S2). The genera *Streptococcus* and *Veillonella* (both from the Firmicutes phylum), *Actinomyces* and *Rothia* (both from the Actinobacteria phylum) and *Prevotella* (from the Bacteroidetes phylum) were the five most abundant in both groups of participants (Fig. 2 and Fig. S1).

### The Inuit oropharyngeal microbiome is distinct from other oropharyngeal microbiomes

**Figure 3:**
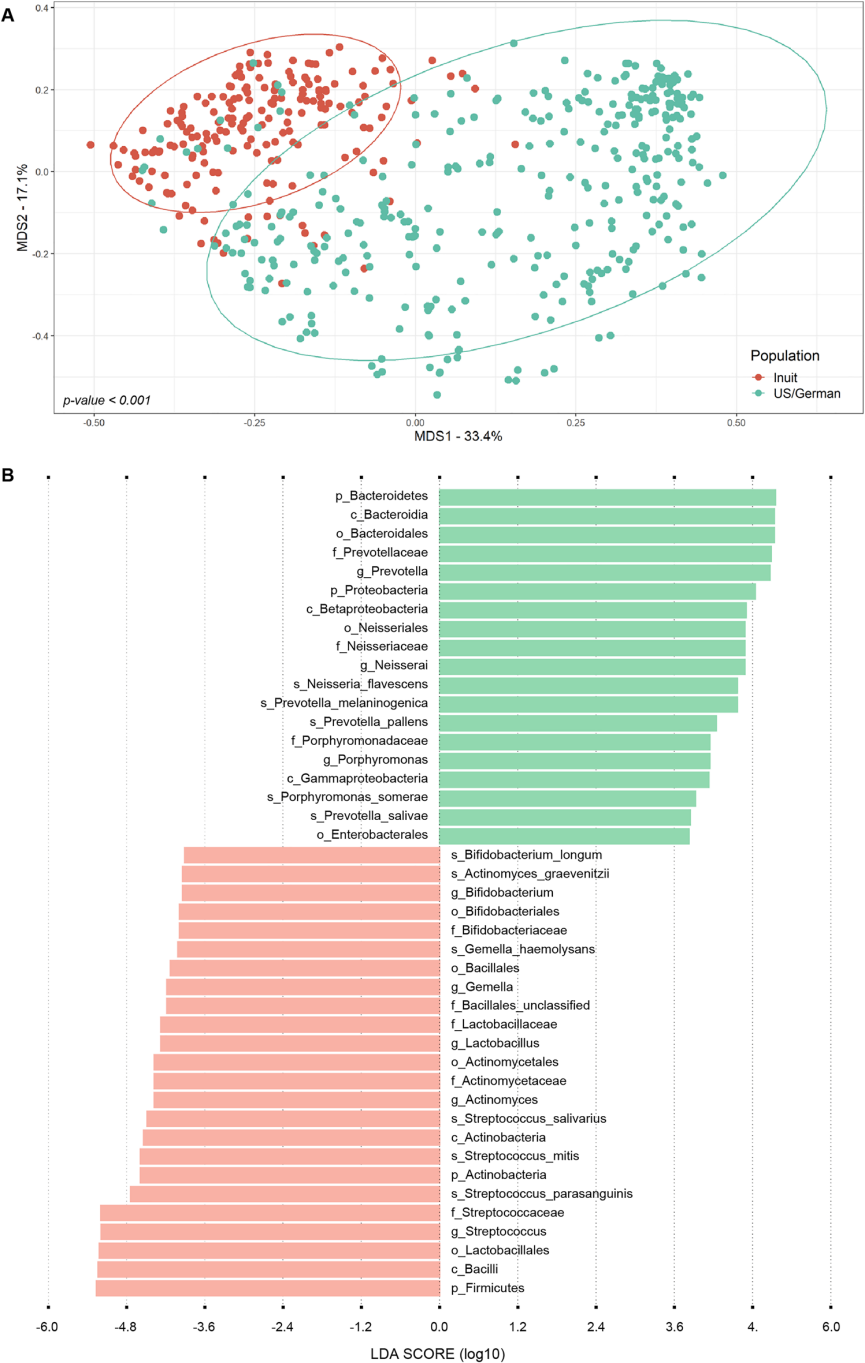
β-diversity metrics and signature taxa of the oropharyngeal microbiome from different populations. (**A**) Clustering of samples from the Inuit (red) and US/German (green) population based on genus-level taxonomic assignments. Clustering is displayed as the non-metric multidimensional scaling (NMDS) plot of all samples, in which the dissimilarity between samples is calculated as the Bray–Curtis distance. The statistical significance of the clustering pattern in the ordination plot was evaluated using the Permutational ANOVA (PERMANOVA) and Analysis of group Similarities (ANOSIM) tests. (**B**) The most significantly discriminant taxa between participants from the Inuit (red) and US/German (green) population were identified by linear discriminant analysis effect size (LEfSe). Taxa with a logarithmic LDA score ≥ 3.8 and *p-value* < 0.001 are shown. *p* phylum, *o* order, *c* class, *f* family, *g* genus, *s* species.

The most abundant phyla, classes and genera observed in the respiratory microbiome of Inuit are consistent with other reports which studied the respiratory microbiome in various populations^17,23,25,26^. We further compared the OP microbiome of the Inuit population with other studies that used a similar approach (OP microbiome, Illumina metagenomic sequencing) and with sequencing data readily available. The studies retained involved US and European (Germany) participants studied in the context of (1) schizophrenia^27^, (2) a *Neisseria meningitidis* outbreak^28^ and (3) critically ill COVID-19 patients^29^, as well as their controls. We also included OP samples archived in the Human Microbiome Project (portal.hmpdacc.org), for a total of 327 OP microbiomes external to the current study. Men and women were included in similar proportion and they aged from 18 and over. The Inuit participants aged from 16 and over. More information on these external samples is given in Table S3. The β-diversity of the Inuit OP microbiomes was significantly distinct (*p-value* = 0.001) and these formed a separate cluster from the US/German samples when visualized by multi-dimensional scaling (Fig. 3A). To exclude bias because of the various diseases, we conducted an analysis with only the control groups of the various studies and found similar cluster separations (Fig. S2) further indicating that the Inuit OP microbiome is indeed distinct. We next investigated the Inuit and US/German OP samples by linear discriminant analysis effect size (LEfSe) at high stringency (LDA = 3.8, *p-value* < 0.001) to determine the features that most likely explain the distinctive nature of the Inuit OP microbiome. We identified signature taxa for both the Inuit and the US/German groups (Fig. 3B). The Inuit group had a higher abundance of two phyla: Firmicutes (including *Veillonella*) and Actinobacteria. Two genera, *Streptococcus* and *Lactobacillus* from the Bacilli class (Firmicutes phylum) were associated to the Inuit group. Another distinctive feature of this group was the enrichment of two genera belonging to the Actinobacteria phylum, *Actinomyces* and *Bifidobacterium*. In contrast, the OP microbiome from the US/German group was enriched in Proteobacteria and notably Gammaproteobacteria and Betaproteobacteria. The genus *Neisseria* belonging to the Betaproteobacteria class was also highlighted as more abundant in this group. Another class, Bacteroidia, with the two genera *Prevotella* and *Porphyromonas* was a considerable feature of US/German group compared to the Inuit group (Fig. 3B).

Similar results were obtained when considering the US and German populations separately. These two populations were distinct from each other and from the Inuit population and clustered separately when visualized by multi-dimensional scaling (Fig. S3A). A SIMPER post-hoc test further revealed that the genera *Actinomyces*, *Bifidobacterium*, *Lactobacillus*, *Gemella* and *Streptococcus*, all part of the most discriminant taxa in the LEfSe described above, were among the 9 shared genera that best explained the dissimilarity between the Inuit and US metagenomes, and between the Inuit and German metagenomes. Importantly, none of these shared genera could explain the dissimilarity between the US and German samples.

### Comparison between the control and bronchial obstruction groups

**Figure 4:**
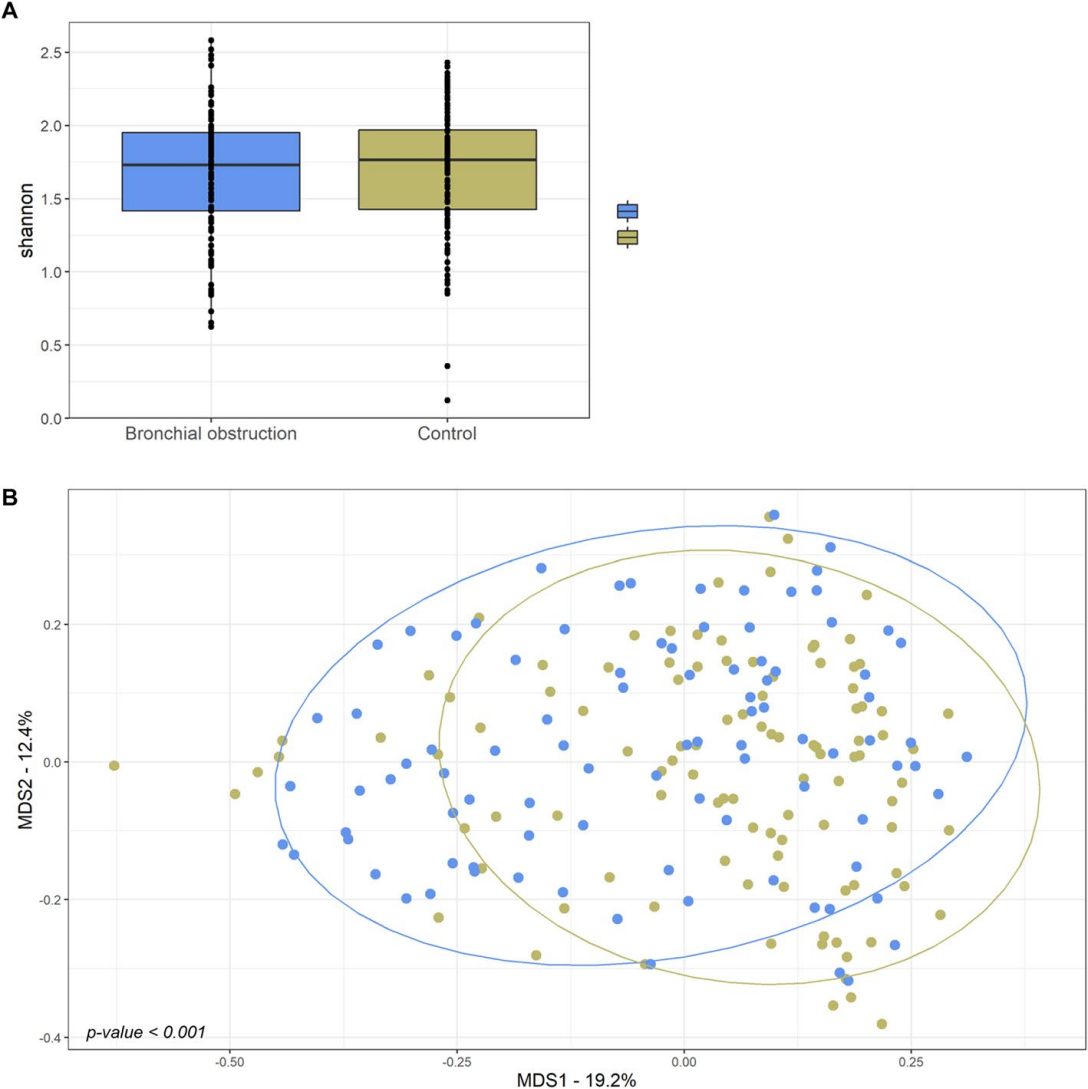
α-diversity and β-diversity metrics of the oropharyngeal microbiome. (**A**) α-diversity measured by the Shannon index for the oropharyngeal microbiomes at the genus level from the bronchial obstruction and the control groups. Each dot denotes the Shannon diversity of a sample. The boxes show inter-quartile ranges for each group with the group’s median denoted by a line. No significant difference was observed between the groups by t-test. (**B**) Clustering of samples from the bronchial obstruction (khaki) and control (blue) groups based on genus-level taxonomic assignments. Clustering is displayed as the non-metric multidimensional scaling (NMDS) plot of all samples, in which the dissimilarity between samples is calculated as the Bray–Curtis distance. The statistical significance of the clustering pattern in the ordination plot was evaluated using the Permutational ANOVA (PERMANOVA) and Analysis of group Similarities (ANOSIM) tests.

We next compared the OP microbiome from the control and bronchial obstruction groups in the Inuit participants to determine whether the microbial community differs according to the presence or absence of bronchial obstruction. We estimated α-diversity to assess the species richness within the OP community of participants from the bronchial obstruction and control groups, and β-diversity to evaluate the similarity of the OP communities between the two groups. No substantial difference in microbial α-diversity was detected between the two groups, whether this was measured by the Chao1, Shannon (Fig. 4A), Simpson or ACE indexes (Fig. S4). Similarly, none of these four α-diversity indexes showed substantial differences between the microbiota derived from participants of the three geographical regions of Nunavik (Table S1 and Fig. S4). However, differences in α-diversity were observed between men and women according to the ACE and Chao1 indexes (Fig. S4C,D), irrespective of their respiratory health status. The α-diversity was also found to decrease as participants were getting older according to the four indices (Fig. S4A–D). According to the abundance-based Bray–Curtis dissimilarity as the distance method, a difference in β-diversity was observed between the control and bronchial obstruction groups (*p-value* = 0.009), suggesting differences in microbial composition (Fig. 4B). This β-diversity was not, however, observed when using the Jaccard coefficient (Fig. S5A). Based on this coefficient, there was a significant difference in β-diversity between men and women (*p-value* = 0.001) (Fig. S5C), as well as between age groups (*p-value* = 0.028) (Fig. S5D).

**Figure 5:**
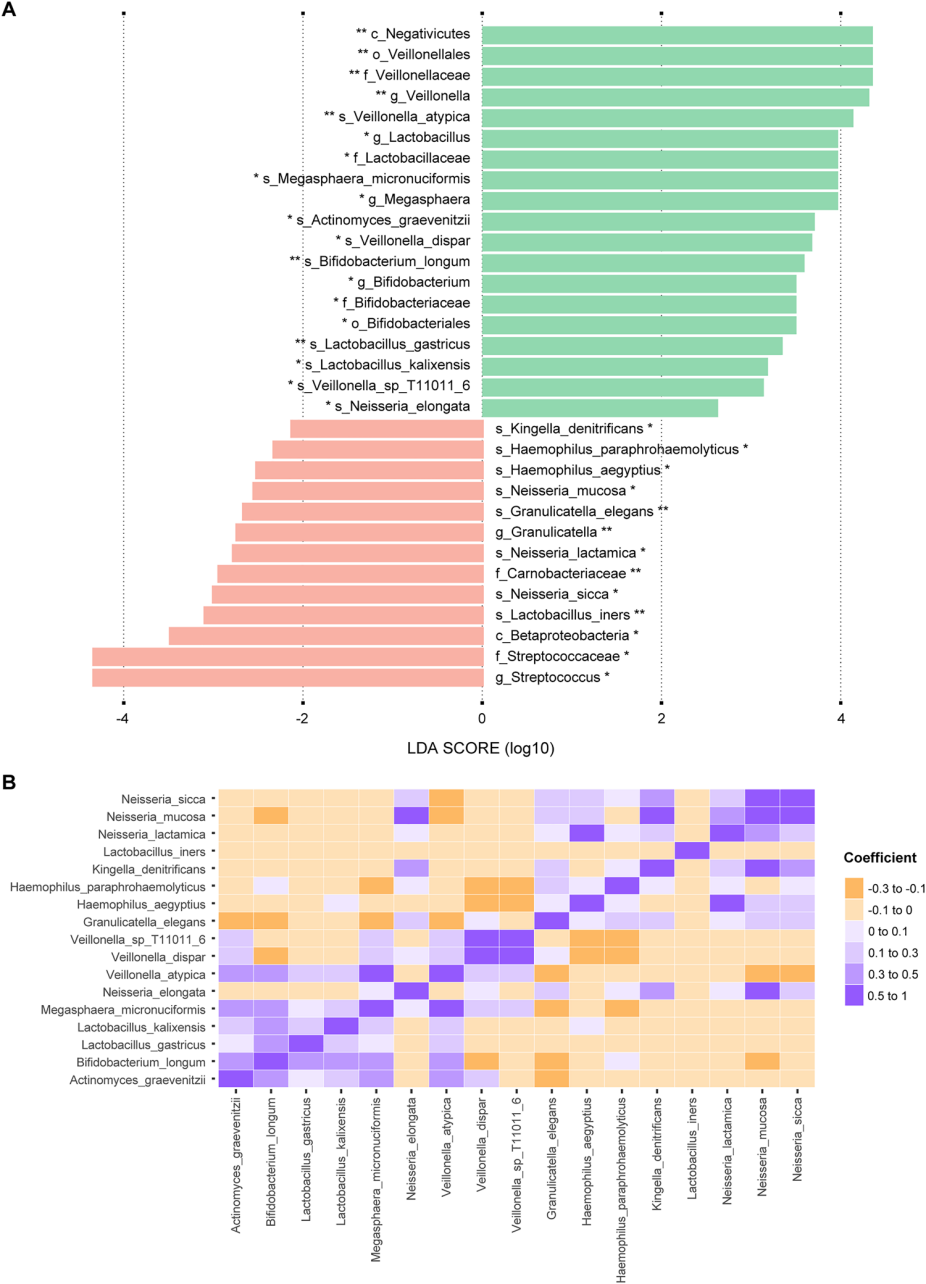
Signature taxa associated with the bronchial obstruction and control groups. (**A**) The most significantly discriminant taxa between participants from the bronchial obstruction (red) and control groups (green) were identified by linear discriminant analysis effect size (LEfSe). Taxa with a logarithmic LDA score ≥ 2.0 and *p-value* < 0.05 (*) or *p-value* < 0.01 (**) are shown. *p* phylum, *o* order, *c* class, *f* family, *g* genus, *s* species (**B**) Heatmap of Pearson’s correlations for the relative abundance of the most discriminant species highlighted by LEfSe analysis in panel A. The orange shaded palette indicates negative correlation while the purple shaded palette indicates positive correlation between species relative abundance.

When looking for taxonomic features responsible for the β-diversity difference between the control and bronchial obstruction groups (Fig. 4B) we found that the Negativicutes, which were among the most abundant bacterial classes in the Inuit group (Fig. 1), were significantly enriched in the control group (*p-value* < 0.01) (Fig. 5A). This is most likely due to the increased abundance in the controls of the genus *Veillonella*, and more specifically of the species *V. atypica* (*p-value* < 0.01) and *V. dispar* (*p-value* < 0.05), as found by LefSe analysis (LDA ≥ 3) (Fig. 5A). The genera *Lactobacillus*, *Megasphaera* and *Bifidobacterium*, along with the species *Megasphaera micronuciformis, Actinomyces graevenitzii, Bifidobacterium longum, Lactobacillus gastricus* and *Lactobacillus kalixensis* were also enriched (LDA ≥ 3, *p-value* < 0.05 or *p-value* < 0.01) in the control group (Fig. 5A). Overall, those species belong in majority to the Firmicutes phylum. In the bronchial obstruction group, an increased abundance was detected for the species *Granulicatella elegans* (as well as its associated *Granulicatella* genus and *Carnobacteriaceae* family) and *Lactobacillus iners* (as well as its associated *Lactobacillus* genus and *Lactobacillaceae* family), both belonging to the Firmicutes phylum (Fig. 5A). The *Streptococcus* genus along with 6 species belonging to the Proteobacteria phylum (including two *Haemophilu*s species), were also more abundant in the bronchial obstruction group (Fig. 2 and Fig. 5A).

We next measured the correlation between the species highlighted by our LEfSe analysis. We found that species enriched in the control group are frequently associated together. For example, *A. graevenitzii*, *B. longum*, *M. micronuciformis*, and *V. atypica* are positively correlated with at least three of the nine species found to also be enriched in the control group (Fig. 5B). Interestingly the presence of *V. atypica* seems to be negatively correlated with at least three species (*G. elegans*, *Neisseria mucosa*, and *Neisseria sicca*) that are enriched in the bronchial obstruction group (Fig. 5B). Species found in the bronchial obstruction group are also more likely to be correlated, with *N. mucosa* associating with four out of eight bacteria that are enriched in that group (Fig. 5B). *N. elongata*, found to be enriched in the control group, is a patent exception as it was not positively correlated with any of the nine other species enriched in the control group, but it was in contrast positively correlated with two species found enriched in the bronchial obstruction group (Fig. 5B). A co-occurrence network generated by Sparse Correlations for Compositional data analysis further reinforced our observation on the correlation between *Neisseria* species, *G. elegans* and *K. denitrificans* in the bronchial obstruction group and between *Veillonella* species, *A. graevenitzii* and *M. micronuciformis* in the control group (Fig. S6).

**Figure 6:**
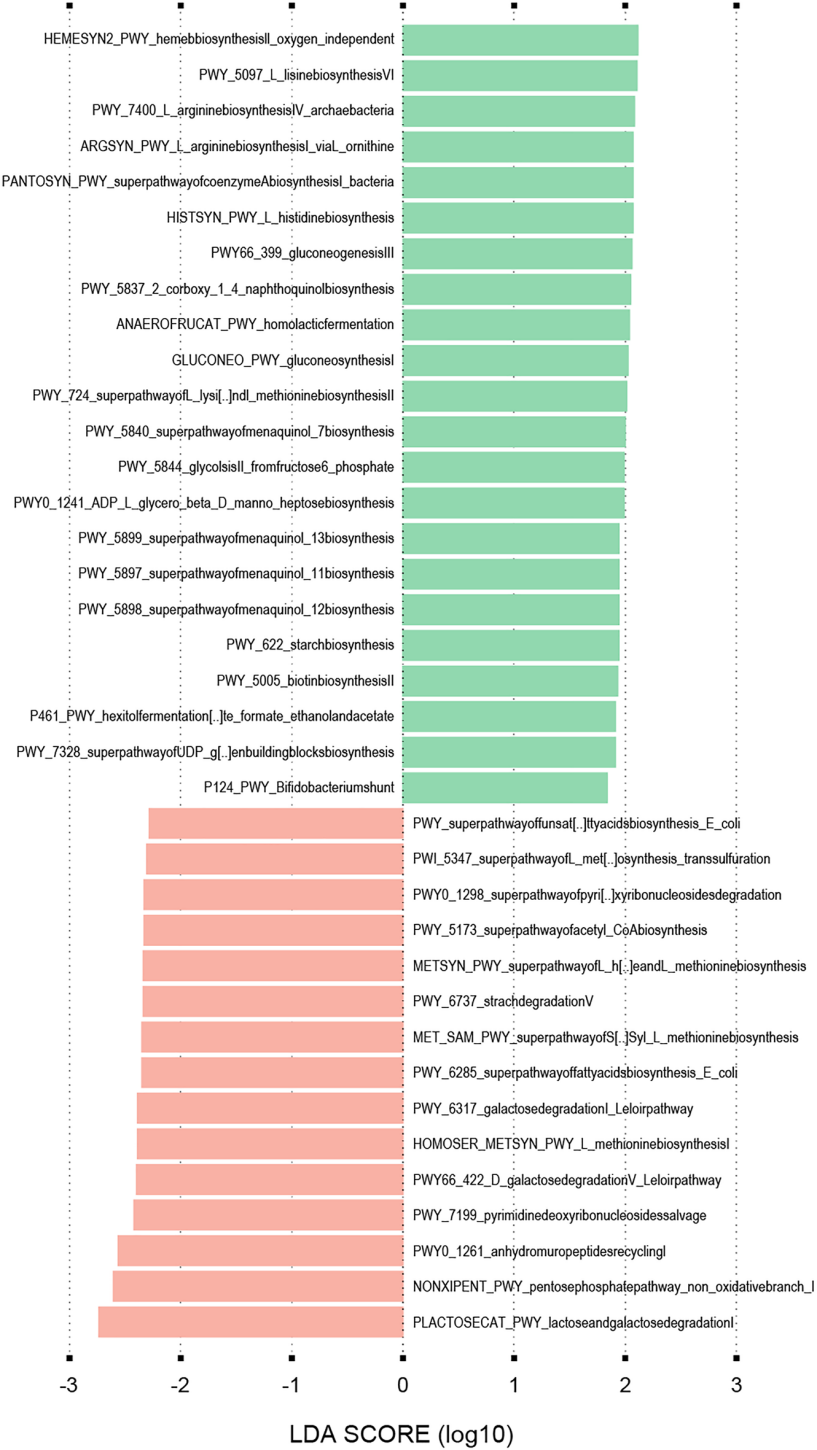
Signature metabolic pathways associated with the bronchial obstruction and control groups. The most significantly discriminant metabolic pathways between participants from the bronchial obstruction (red) and control groups (green) as identified by linear discriminant analysis effect size (LEfSe).

Our approach of whole metagenome sequencing has facilitated the analysis of metabolic functions putatively linked to respiratory health. This was carried out using the HUMAnN 3.0 pipeline and followed by LEfSe analysis with default parameters (LDA = 2, *p-value* < 0.05). Four pathways of biosynthesis of menaquinone (vitamin K2) derivatives with different numbers of isoprene units in their side chains (PWY-5840, PWY-5897, PWY-5898, PWY-5899) were enriched in the control group (Fig. 6). Intriguingly, two other related pathways were also enriched in the control group: (i) The pathway of biosynthesis of 2-carboxy-1,4-naphthoquinol (PWY-5837), which is a branch point metabolite leading to the biosynthesis of menaquinone and (ii) A heme biosynthesis pathway (HEMESYN2-PWY), which requires menaquinone for the synthesis of the heme precursor protoporphyrin IX (Fig. 6). Enrichment of other vitamin-related pathways were also detected in the control group, such as the biotin (vitamin B7) biosynthesis pathway (PWY-5005) and the super pathway of coenzyme A biosynthesis (PANTOSYN-PWY), which involves the biosynthesis of pantothenate (vitamin B5) (Fig. 6). Coenzyme A is a cofactor that plays a key role in energy metabolism as found on MetaCyc database (biocyc.org/META). Many pathways providing cellular energy were also increased in the control group including ATP synthesis (PWY-7219), gluconeogenesis (PWT66-399, GLUCONEO-PWY), glycolysis (PWY-5484) and lactic fermentation (ANAEROFRUCAT-PWY), including a variation (P124-PWY) of the latter found mostly in the *Bifidobacterium* genus (Fig. 6). We also noticed an increase in pathways involved in the biosynthesis of positively charged amino acids (PWY-5097, PWY-7400, ARGSYN-PWY, HISTSYN-PWY) and in methionine (PWY-724) although alternate methionine biosynthesis pathways (PWY-5347, MET-SAM-PWY, HOMOSER-METSYN-PWY) were also increased in the bronchial obstruction group (Fig. 6). In the latter group we detected increased pathways involved in fatty acid biosynthesis (PWY-6284 and 6285), as well as degradation of complex sugars (PWY-6317, PWY-6737, PWY66-422, LACTOSECAT-PWY) (Fig. 6).

### Convergence of biological findings through alternative bioinformatic analyses

While MetaPhlAn3 is widely used for microbiome analysis, comparison with other algorithms is warranted in light of some of reported differences in microbiome analysis that are software-dependent^30,31^. We reinvestigated the whole metagenome data set by using the computational tool mOTUs2.0. This tool highlighted 502 species and the most abundant phyla observed were similar to the analysis done with MetaPhlAn3, with Firmicutes dominating (Fig. S7). Consistent with the MetaPhlAn3 analysis, *Streptococcus, Veillonella*, *Actinomyces, Rothia*, and *Prevotella* were the 5 most abundant genera observed by mOTUs2.0 (Fig. S1). When we carried out a LEfSe analysis at LDA ≥ 3 *(p-value* < 0.01) the output derived from MetaPhlAn3 and mOTU2 was highly similar, with *Veillonella* (as a genus) and *V. atypica*, *B. longum*, and *L. gastricus* found to be enriched in the control group (Fig. S8). mOTUs2 also highlighted *Atopobium parvum* and *V. tobetsuensis* as enriched in the control group (Fig. S8). The genus *Granulicatella* was found by both tools as enriched in the bronchial obstruction group (Fig. S8).

**Figure 7:**
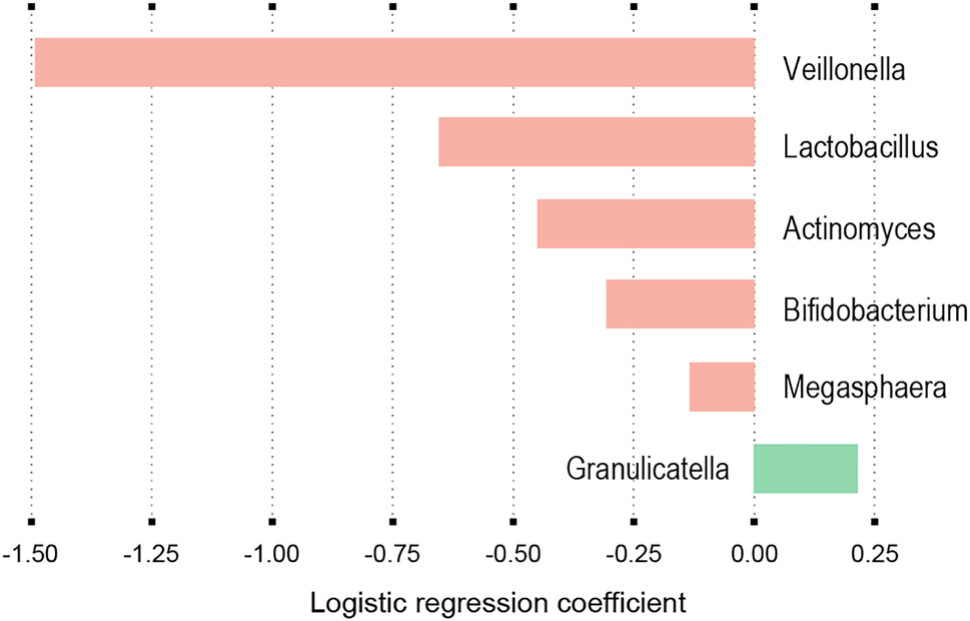
Machine learning models to predict healthy and bronchial obstruction groups. The best model using MetaPhlAn3 genus relative abundance as input was obtained with the features selected by the Wilcoxon-test and a Logistic regression model with an accuracy score of 63.1%. Since the bronchial obstruction group was considered as the positive class, a positive coefficient means an increase and a negative coefficient a decrease in the probability of having obstructive syndrome. The strength of the effect is represented by the magnitude of the coefficient.

We also attempted to discriminate the control group from the bronchial obstruction group by investigating MetaPhlAn3, mOTUs2 and HUMAnN3 outputs with a series of machine learning (ML) algorithms and feature selection methods (see “Material and methods” and Table S4). Our highest classification accuracy (76%) was achieved, after hyperparameter tuning, by the model using mOTUs2 species relative abundance as input, Chi-squared test as feature selection method and decision tree as classification algorithm (Table S4). We next examined the features retained by the best model for each microbiome data type (Supplementary Data File). Interestingly, many of those features were also highlighted in the LEfSe analysis. For instance, the best model using MetaPhlAn3 genus relative abundance as input selected *Veillonella*, *Lactobacillus*, *Actinomyces*, *Bifidobacterium* and *Megasphaera* as negative predictors of bronchial obstruction, and *Granulicatella* as positive predictor of bronchial obstruction (Fig. 7) consistent with our LEfSe analysis (Fig. 5).

## Discussion

We tallied a large respiratory health survey of the Inuit population of Nunavik^6^ by a comprehensive OP microbiome study. The survey indicated that airway obstruction (17% vs. 12%) and diagnosed COPD (14% vs. 9%) but not diagnosed asthma (3% vs. 15%) were more frequent in Nunavik compared to the rest of Canada (numbers age-adjusted based on the Nunavik population)^6^. The results derived from this recent survey are in line with previous studies^2,5^. These prevalence rates must be considered in the context that participants had to travel to an icebreaker in order to be evaluated for the health survey, possibly reducing the possibility for individuals with severe bronchial obstruction to participate. Yet we had a large group (n = 198) of Nunavimmiut OP swabs with linked spirometry values that provide indication about respiratory health and the presence of bronchial obstruction.

The overall Inuit OP microbiome composition was consistent with other studies where the four predominant phyla found here (Firmicutes, Actinobacteria, Bacteroidetes and Protobacteria) were also reported either in healthy individuals or in people with various respiratory diseases^13,16,23,32^. *Streptococcus* was the most abundant genus in our samples, a finding consistent with its predominance in oral, bronchial and lung tissues^13^. In addition to *Streptococcus,* we found that *Veillonella, Actinomyces, Rothia* and *Prevotella* made up the 5 most abundant genera. These results are reproducible as they were replicated by two distinct taxonomy profilers (MetaPhlAn3 and mOTUs2). These five genera are indeed considered to be part of the core pulmonary microbiome^33,34^ and are thought to be pivotal for a healthy respiratory flora^16,35^. While the most abundant classes and genera in our Inuit group were alike other OP microbiomes, an analysis of similarities revealed that they nonetheless had distinctive features. The Inuit were indeed richer with the phyla Firmicutes and Actinobacteria, while US/German groups were richer in Bacteroidetes and Proteobacteria. Firmicutes and Actinobacteria are predominantly associated with respiratory health (in contrast to Proteobacteria) and their abundance in the Inuit may reflect the relatively good respiratory health of this population despite high smoking rates^6^. Diet and lifestyle have been shown to influence the gut microbiome^15,19^ which is distinct between industrialized, non-industrialized and Nunavik populations^36^. Traditional Inuit foods, which mostly includes meat from hunted species and fish, may affect to some extent the Inuit gut microbiome, which itself may influence the OP microbiome through the gut-lung axis^37^.

It is important to acknowledge that a possible limitation of our comparison of Inuit and non-Inuit OP microbiomes pertains to the sample preparation methods of the different studies involved (Table S3), as sample preparation may introduce biases in taxon levels. However, sample preparation methods most likely had no impact here and should not affect our conclusions. Indeed, samples from different studies are intertwined with Inuit samples in an NMDS plot colored by sample prep kits (Fig. S3B). More importantly, the separation of Inuit samples in the NMDS plot remains when the analysis is restricted to external samples prepared with the same kit as we used (Fig. S3C). The sequencing platform used by the different studies also had no impact (Fig. S3D).

Alterations in microbial richness or composition are usually observed with severe COPD^12,17,26,33^ or bronchiectasis^38^. In our analysis we did not find a difference in α-diversity based on the respiratory health of our participants. One possible explanation is that samples from our bronchial obstruction group were derived from people with only mild or moderate bronchial obstruction whose OP microbiomes do not display a significantly different α-diversity^33^. While not the norm, some studies reported no differences in α-diversity between people with and without respiratory disease^8,39,40^. However, we were able to find differences in α-diversity metrics when we stratified groups according to sex, women having a less diverse microbial flora. Diseases of the respiratory systems were found to be higher in Inuit females^2,41,42^. This difference in α-diversity in the OP microbiome between men and women was significant and consistent with studies in other populations^43,44^. The OP microbiome of mid-aged adult and elders were found to be different^45^, a finding consistent with our observation that α-diversity decreases with age.

While no difference in microbial α-diversity could be detected between our two spirometry groups, their microbial composition was significantly different according to β-diversity metrics. Such difference in microbial composition was also observed when considering sex and age. Overall, a variety of genera and species part of the Firmicutes phylum were increased in the control group. *Veillonella*, a member of the core pulmonary bacterial microbiome^16,33^ was also increased in the control group, including all its taxonomic affiliations (family, order, class, phylum). This genus, notably the species *V. dispar* and *V. atypica*, stands out as being consistently associated with healthy oral microbiota^35^ and preserved lung function^18,34^. Other Firmicutes of the Bacilli (e.g. the *Lactobacillus* genus) and Negativicutes (e.g. the *Megasphaera* genus) classes were also increased in the control group. Several *Lactobacillus*, including *L. gastricus*, are potential probiotic candidates^46^ and *M. micronuciformis* seems beneficial to health^47^ and is often associated with *Veillonella*^48^. The other phylum increased in the control group was the Actinobacteria (e.g. the genera *Actinomyces* and *Bifidobacterium*). Many reports have shown, at least in murine models, that *B. longum* can protect against lung inflammation and can decrease the severity of airway disorders^49^. These data are meaningful as a similar enrichment was observed when we used the mOTUs2 algorithm instead of MetaPhlAn3. Moreover, through a logistic regression model of machine learning, the same species were predictive of respiratory health. Species found in the control group are also more likely to be correlated together. *Neisseria elongata* was the exception but it had the lowest LDA score and may thus have been a false positive in the LefSe analysis.

In the bronchial obstruction group, diverse genera and species part of the Proteobacteria phylum (e.g. *Kingella*, *Haemophilus*, *Neisseria*) and the Bacilli class (e.g. *Granulicatella*) were enriched. Two *Haemophilu*s species were enriched in participants from this group. Our data were also in line with frequent bacterial genera described in COPD patients, for example the *Haemophilus*, *Streptococcus* and *Neisseria* genera associated here to participants with impaired lung function^9,13,14,50^. Similarly, increased levels of Carnobacteriaceae have been associated with respiratory infections in a pneumonia mouse model^51^. *Granulicatella elegans*, which belongs to this bacterial family, was increased (*p-value* < 0.01) in our bronchial obstruction group and while part of the normal flora, this bacteria has been shown to induce mediators of inflammation^52^. Notably, the latter was also found to be increased by mOTUs2, a feature associated with bronchial obstruction in our logistic regression machine learning model. *Haemophilus* spp. and *Neisseria* spp. were also part of the main features of our machine learning model with the best score.

A number of biochemical pathways related to vitamin metabolism were enriched in the analysis of the OP microbiome of the control group (menaquinone, biotin and the pantothenate containing coenzyme A). The vitamin biosynthetic pathways are in general depleted across groups with various chronic diseases in comparison to healthy controls^53^. Menaquinone (vitamin K2) is proposed to play a distinct role in the prevention of multiple diseases, while biotin is linked to microbial and host metabolism as well as to inflammation^54^. Reduced inflammation mediated by bariatric surgery was associated with increased bacterial biotin^55^. Coenzyme A is an enzymatic co-factor involved in a plethora of metabolic reactions and the increase of one of those enzymes involved in butyrate metabolism is linked to asthma protection^56^. Fermentation pathways (ANAEROFRUCAT-PWY, P461-PWY) were also increased in our healthy controls, with the possibility of producing short chain fatty acids that are linked to anti-inflammatory effects^54^. In the bronchial obstruction group, we detected distinct enriched pathways that are involved in long chain fatty acid biosynthesis and in complex sugar catabolism that could impact negatively on lung function^57^, increasing the risk of respiratory diseases^58^.

In summary, taking advantage of a large survey of respiratory health, we have carried out a detailed OP microbiome analysis of the Inuit population of Nunavik. We found that the Inuit OP microbiome was distinct in comparison to other populations. In the context of respiratory health, we also found differences in bacterial taxonomic groups between healthy controls and the group with mild to moderate bronchial obstruction. Through two different algorithms and further supported by a machine learning strategy, we found that *Veillonella* and other Firmicutes were more abundant in healthy controls, while Proteobacteria and *Graniculatella* were enriched in the bronchial obstruction group. These associations, which are consistent with other studies in completely different populations, suggest potential microbiological ecological strategies for improving respiratory health.

## Methods

### Ethic statement and sampling

This study was approved by the Research Ethical Committee of Laval University Hospital center, Québec, with project number MP-20-2019-4110 pertaining to the 2017 Qanuilirpitaa? survey. All methods were performed in accordance with the relevant guidelines and regulations. This survey was a broad health survey which include a comprehensive respiratory part into it. Participants were enrolled and sampled in the period between August 19, 2017 and October 5, 2017 in 14 villages from three regions of Nunavik—Hudson Bay, Ungava Bay and Hudson strait. All participants were recruited with informed consent for the use of their anonymized data in research. OP samples were obtained by research staff that rubbed a sterile flocked swab at the back of the subject’s throat in accordance with the guidelines from the medical microbiology laboratory of the Centre Hospitalier Universitaire de Québec. The swabs were put in Universal Transport Medium immediately upon sampling and kept at 4 °C for no longer than 30 min prior to be stored at − 80 °C. Swabs were then kept frozen at − 80 °C until further processing. Also, spirometry was performed by experienced respiratory therapists using an EasyOneTM Spirometer (New Diagnostic Design, Andover, MA, USA), and following the protocol from the CanCOLD study^59^. Two lung volumes were measured: forced vital capacity (FVC) and forced expiratory volume in one second (FEV_1_). All spirometric tracings were reviewed for validity by a respirologist. All participants with at least three acceptable spirometry curves were included in the analysis^60^. Bronchial obstruction was defined by a FEV_1_/FVC ratio below 0.7, a standard value used to compare the lung function of many ethnic groups in different countries^24^.

### Extraction and sequencing

Throat swabs were thawed on ice and vortexed for 10 s. DNA extraction from the swabs was performed using the QIAamp DNA Microbiome kit (QIAGEN) according to the manufacturer’s instructions. We used a Mini-Beadbeater-24 (BioSpec Products) for mechanical lysis, applying 3500 oscillations per minute 3 times for 2 min with an iced-storage 5-min interval. Lysates were incubated at 56 °C for 30 min. For elution, we used 50 µl of buffer AVE which was eluted twice per column. The extracted DNAs were quantified using a Quantus™ Fluorometer (Promega) and stored at − 20 °C. Next generation sequencing libraries were made using the Nextera DNA Flex Library Prep kit (Illumina) following the manufacturer’s instructions. Libraries were quantified using a Quantus™ Fluorometer, and quality was checked with an Agilent 2100 Bioanalyzer with High Sensitivity DNA chips (Agilent). As a control we used mocked swabs and Universal Transport Medium spiked with 10^5^ CFU of *Pseudomonas aeruginosa* (ATCC 27853) and *Acinetobacter baumanii* (ATTC 19606) that were processed similarly as above. Sequencing of the libraries was done with an Illumina NovaSeq 6000 system, generating approximately 100-million paired-end reads of 150 nucleotides per sample. Sequencing of the mocked library revealed only the spiked bacteria, suggesting that contamination was marginal.

### Bioinformatics

Trimming of paired-end sequencing reads was done using Trimmomatic. To quantify the percentage of human DNA contamination in our sample, we aligned the reads to the Genome Reference Consortium Human Build 38 patch release 13 (https://www.ncbi.nlm.nih.gov/assembly/GCF_000001405.39/) using Bowtie2^61^. For taxonomic profiling, sequence reads from each sample were mapped against the MetaPhlAn3 clade-specific marker gene database mpa_v30_CHOCOPhlAn_20190 using MetaPhlAn3^30^. For metabolic profiling, we used the HUMAnN3 (v3.0.0.α.1)^30^ and the databases uniref90 and mpa_v30_CHOCOPhlAn_201901^30^. All pathways assigned as “unclassified”, “unintegrated” or “unidentified” were excluded from subsequent analyses. Taxonomic profiles were also generated using the mOTUs2 profiler version 2.0.0^62^ at the species, genus, family, and class taxonomic levels using default parameters. For comparison between Inuit and US/German populations, reads were taken from three databases: 1. Sequence Read Archive—SRA (ncbi.nlm.nih.gov/sra); 2. European Nucleotide Archive—ENA (ebi.ac.uk/ena); 3. Human Microbiome Project (hmpdacc.org). Criteria selection were shotgun sequencing and OP swabs from participants over 16 years of age. These were analyzed using the same MetaPhlAn3 pipeline as for the Inuit samples. Given that we observed a correlation between the number of reads per sample and the number of genera identified, samples with less than 5 genera and with ≥ 90% of unassigned reads were excluded from downstream analyses, to avoid possible biases coming from low sequencing coverage. The correlation between low read counts and the number of genera was no longer observed upon sample exclusion. A total of 327 US/German samples were kept for analysis. All Inuit samples satisfied the exclusion criteria.

### Statistical analysis

Species relative abundances obtained from MetaPhlAn3 were imported into R v4.0.4 and α-diversity analyses were performed with the vegan 2.5-7 (Shannon and Simpson), fossil 0.4.0 (ACE) and breakaway 4.7.3 (Chao1) packages^63–65^. We assessed statistical differences of these four measures between more than two groups by Analysis of Variance (ANOVA) and by student’s t-test for two-group comparisons. Post-hoc pairwise group comparisons for more than two groups were performed with Tukey-HSD (Honest Significant Differences) tests. For β-diversity, Bray–Curtis dissimilarity and Jaccard index were calculated using the vegan package and PCoA plots were drawn using the ggplot 2 3.3.5 package. Analysis of similarities (ANOSIM) and Permutational ANOVA (PERMANOVA) was used to compare β-diversity measures. Post-hoc comparisons were made with the SIMPER function from the vegan package in R^66^. Significant differences between groups, for both taxonomic affiliation and metabolic pathways, were depicted by LEfSe analysis using Kruskal–Wallis rank sum and Wilcoxon tests (α = 0.05) with LDA threshold fixed at 2^67^. For some analysis which required more stringency, the alpha-value was fixed at 0.01 or 0.001 and LDA threshold at 3.8. Pearson correlation coefficient was calculated with ggpubr v0.4.0 package in R^68^. The Sparse Correlations for Compositional data analysis^69^ was performed using the NetCoMi R package^70^. For ease of visualization, we focused on the 17 species highlighted by the LEfSe analysis comparing the bronchial obstruction and control groups of the Inuit dataset.

### Machine learning

We formulated the discrimination between two groups (bronchial obstruction vs control) as a binary classification task, the obstructive group was regarded as the positive class and the control group as negative. We investigated three different microbiome data types, including MetaPhlAn3/mOTUs2 taxa relative abundance (species/genus/family levels) and HUMAnN3 pathway relative abundance. Data were normalized by scaling their values in the range from 0 to 1. Ten ML algorithms were explored including Logistic regression, Decision tree, k-Nearest neighbors, Support vector machines, Linear discriminant analysis, Random forest, Adaptive boosting (AdaBoost), Bagging, Extremely randomized trees and Gradient boosting. Nine feature selection methods were evaluated, namely the t-test, the Wilcoxon rank-sum test, the Mann–Whitney U test, the Chi-squared test, the F-test, the Mutual information, the Logistic regression, the LASSO (Least Absolute Shrinkage and Selection Operator) and the Random forest. For the t-test, the Wilcoxon rank-sum test and the Mann–Whitney U test methods, we used a *p-value* cut-off of 0.05 to select features for the next step. For the Chi-squared test, the F-test and the Mutual information methods, top 10, 20 and 30% of highest scoring features were selected. For the Logistic Regression and the Random Forest methods, features whose importance is greater or equal to the mean of the feature importance were kept. For the LASSO method, the threshold used is 10^–5^. The feature selection step and the learning step were wrapped in a pipeline to prevent data leakage. Hyperparameter tuning was performed using grid search. The predictive performance of each model was accessed by leave-one-out cross-validation. Machine learning models were implemented using Scikit-learn library (version 1.0.1)^71^.

### Supplementary Information

Supplementary Information 1.

Supplementary Information 2.

## Data Availability

The next generation sequencing dataset that support the findings of this study were used under copyright agreement and are available upon request. It is stored on the VALERIA data management platform of Université Laval (https://valeria.science/) under controlled access. In accordance with the First Nations principles of ownership, control, access, and possession (OCAP®), the Nunavik Regional Board of Health and Social Services is the owner of the data and of the biological samples collected during the Qanuilirpitaa? 2017 health survey on behalf of the Inuit population of Nunavik. Any request for data access should be addressed to the Qanuilirpitaa? 2017 Data Management Committee (nunavikhealthsurvey@ssss.gouv.qc.ca) that oversees the management of the survey data as well as its biological samples and digital data. This management framework is integral to the research partnership process and is intended to allow researchers to access and use the data in a manner that is respectful of all parties. Additional information on data ownership, management, and access, as well as the Data/Biological Samples Request and Analysis Proposal Application Form are available in the Methodological report of the Qanuilirpitaa? 2017 (see Appendix ‘Policy on the management of databases and biological samples’, Section 7, pages 14–16 (415–417) and Appendix B, pages 23–27 (424–428); https://nrbhss.ca/sites/default/files/health_surveys/A11991_RESI_Rapport_methodologique_EP4.pdf).
