# Supplementary material for: Distinctive features of the oropharyngeal microbiome in Inuit of Nunavik and correlations of mild to moderate bronchial obstruction with dysbiosis
Source: Sci Rep. 2023 Oct 3;13:16622. doi: 10.1038/s41598-023-43821-4 (PMC10547696; doi:10.1038/s41598-023-43821-4)
Supplement: Supplementary file 1 — Supplementary Information 1. [file 41598_2023_43821_MOESM1_ESM.pdf]

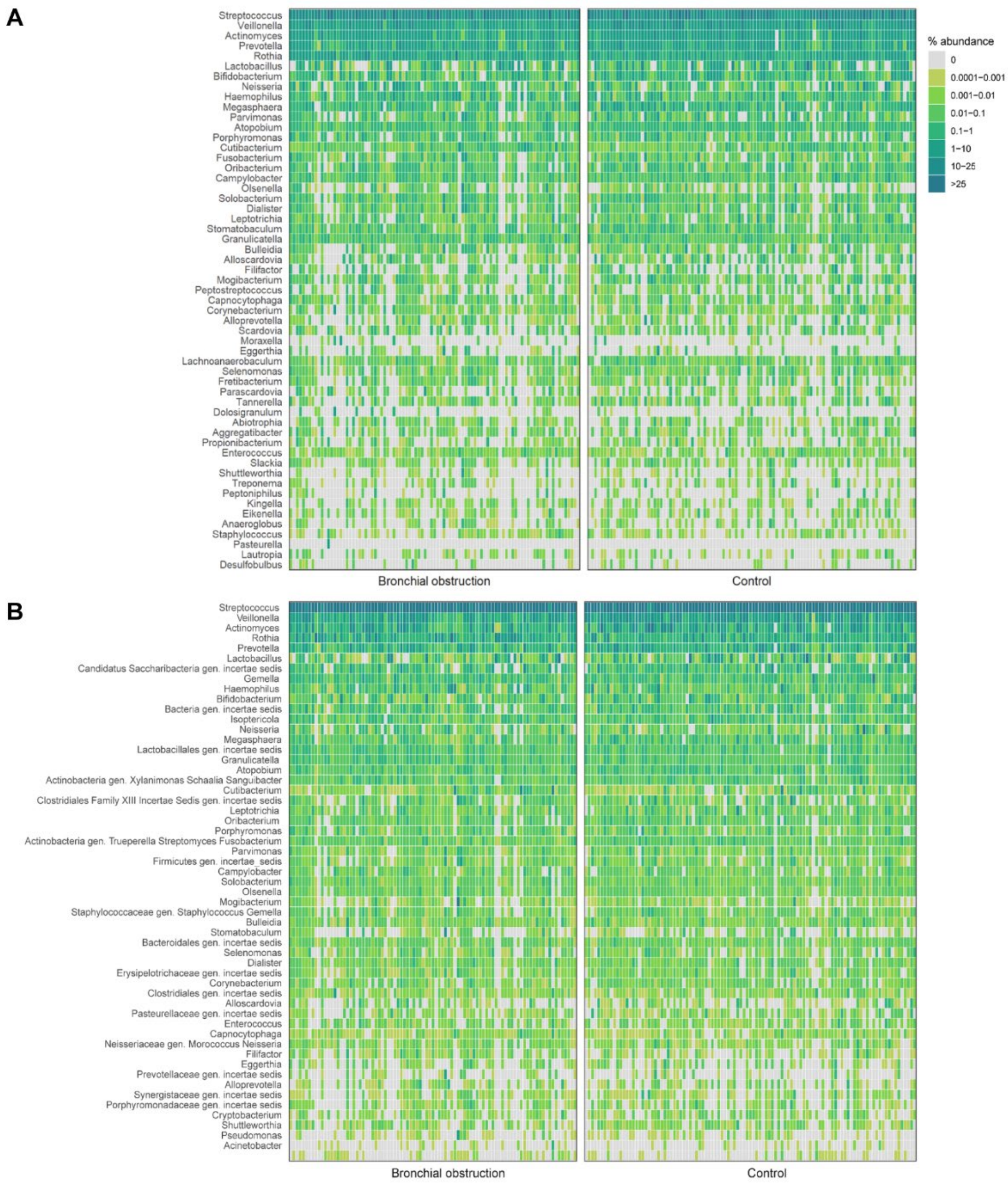

Supplementary figure 1. Most abundant bacterial genera among participants. The heatmap shows the relative abundance of the 55 most abundant bacterial genera detected in the bronchial obstruction and control groups as obtained with MetaPhlAn3 (A) and mOTUs2 (B). Bacterial genera were included if the sum of their relative abundance in all samples was greater than 0.8%. For uniformity with Metaphlan3, the mOTUs2 analysis was restricted to reference marker gene-based genera (i.e. metagenomic marker gene-based taxonomic units were excluded).

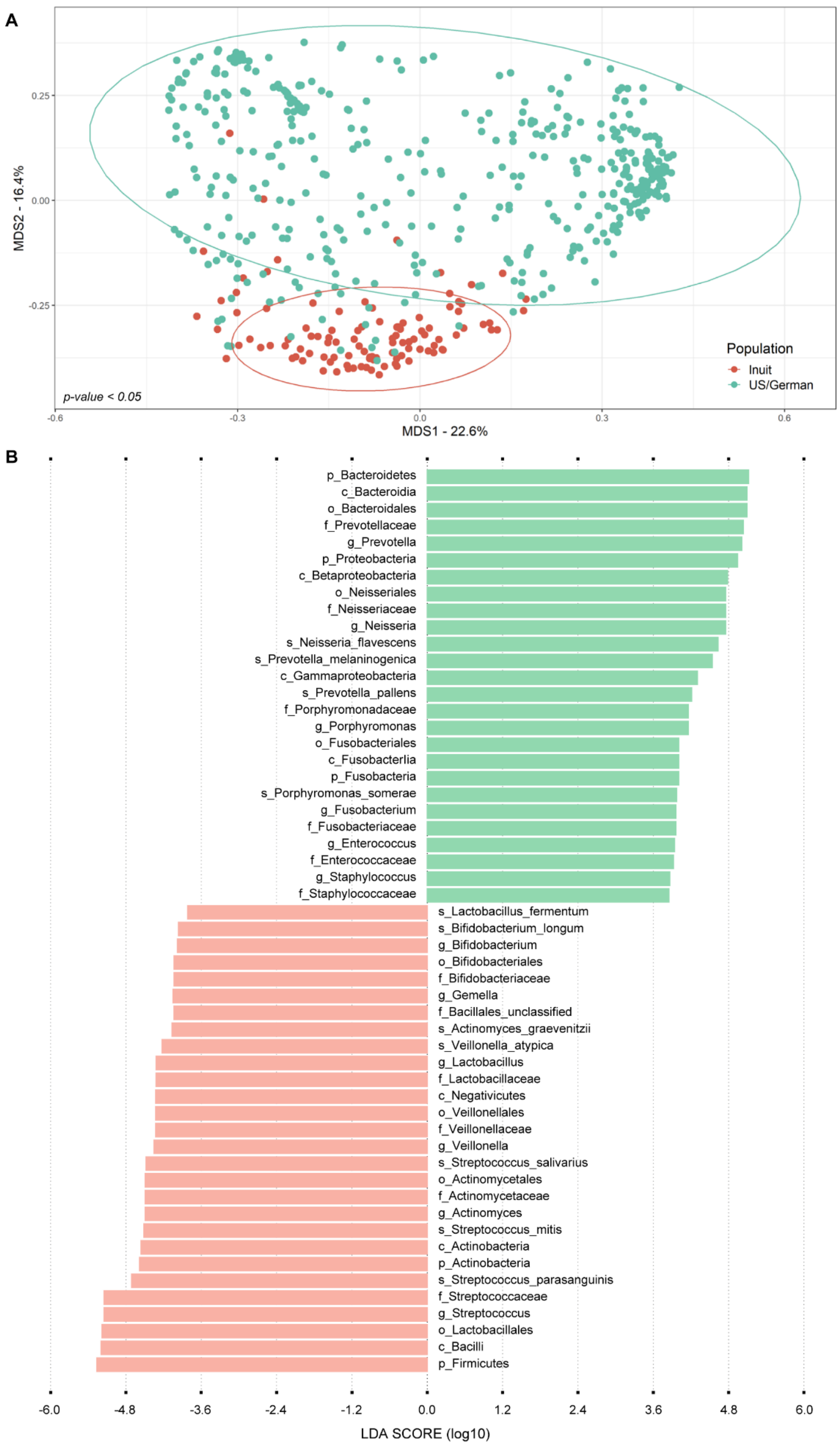
A
B
MDS2 - 16.4%
MDS1 - 22.6%
Population
Inuit
US/German
p-value < 0.05
0.25
0.00
-0.25
-0.6
-0.3
0.0
0.3
0.6
p_Bacteroidetes
c_Bacteroidia
o_Bacteroidales
f_Prevotellaceae
g_Prevotella
p_Proteobacteria
c_Betaproteobacteria
o_Neisseriales
f_Neisseriaceae
g_Neisseria
s_Neisseria_flavescens
s_Prevotella_melaninogenica
c_Gammaproteobacteria
s_Prevotella_pallens
f_Porphyromonadaceae
g_Porphyromonas
o_Fusobacteriales
c_Fusobacterlia
p_Fusobacteria
s_Porphyromonas_somerae
g_Fusobacterium
f_Fusobacteriaceae
g_Enterococcus
f_Enterococcaceae
g_Staphylococcus
f_Staphylococcaceae
s_Lactobacillus_fermentum
s_Bifidobacterium_longum
g_Bifidobacterium
o_Bifidobacteriales
f_Bifidobacteriaceae
g_Gemella
f_Bacillales_unclassified
s_Actinomyces_graevenitzii
s_Veillonella_atypica
g_Lactobacillus
f_Lactobacillaceae
c_Negativicutes
o_Veillonellales
f_Veillonellaceae
g_Veillonella
s_Streptococcus_salivarius
o_Actinomycetales
f_Actinomycetaceae
g_Actinomyces
s_Streptococcus_mitis
c_Actinobacteria
p_Actinobacteria
s_Streptococcus_parasanguinis
f_Streptococcaceae
g_Streptococcus
o_Lactobacillales
c_Bacilli
p_Firmicutes
-6.0
-4.8
-3.6
-2.4
-1.2
0.0
1.2
2.4
3.6
4.8
6.0
LDA SCORE (log10)

Supplementary figure 2. **β-diversity metrics of the oropharyngeal microbiome from control group of different populations**. Clustering of samples from the control groups of Inuit (red), US (purple), and German (green) populations based on genus-level taxonomic assignments. Clustering is displayed as the non-metric multidimensional scaling (NMDS) plot of all samples, in which the dissimilarity between samples is calculated as the Bray-Curtis distance. The statistical significance of the clustering pattern in the ordination plot was evaluated using the Permutational ANOVA (PERMANOVA) and Analysis of group Similarities (ANOSIM) tests.

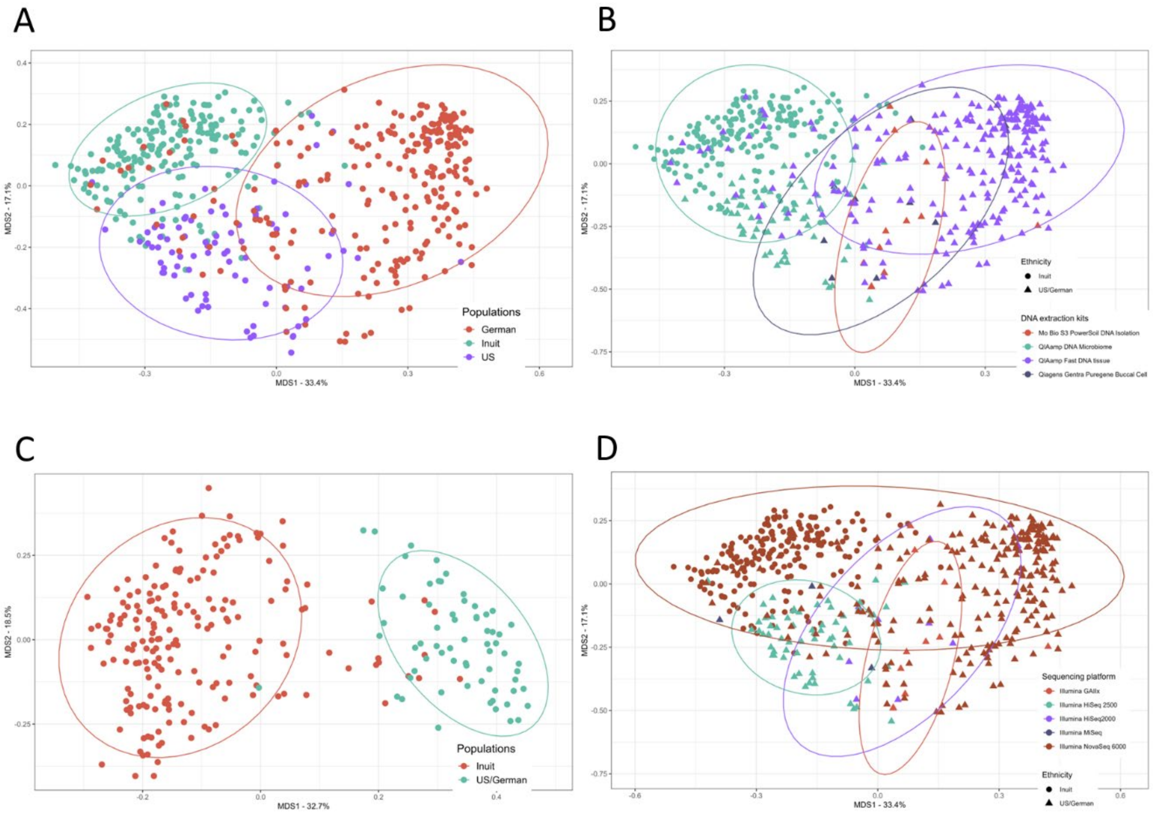

Supplementary figure 3. β-diversity metrics of the oropharyngeal microbiome from the three different populations. (A) Clustering of samples from the Inuit (green), US (purple) and German (red) populations based on genus-level taxonomic assignment. (B) Clustering of samples based on genus-level taxonomic assignment colored by sample preparation kits and shaped by ethnicity. (C) Clustering by genus-level taxonomic assignment for samples prepared with the QIAamp DNA Microbiome kit, colored by ethnicity (Inuit, red; non-Inuit, green). (D) Clustering of samples based on genus-level taxonomic assignment colored by sequencing platforms and shaped by ethnicity. For all plots, clustering is displayed as the non-metric multidimensional scaling (NMDS) plot of all samples, in which the dissimilarity between samples is calculated as the Bray-Curtis distance.

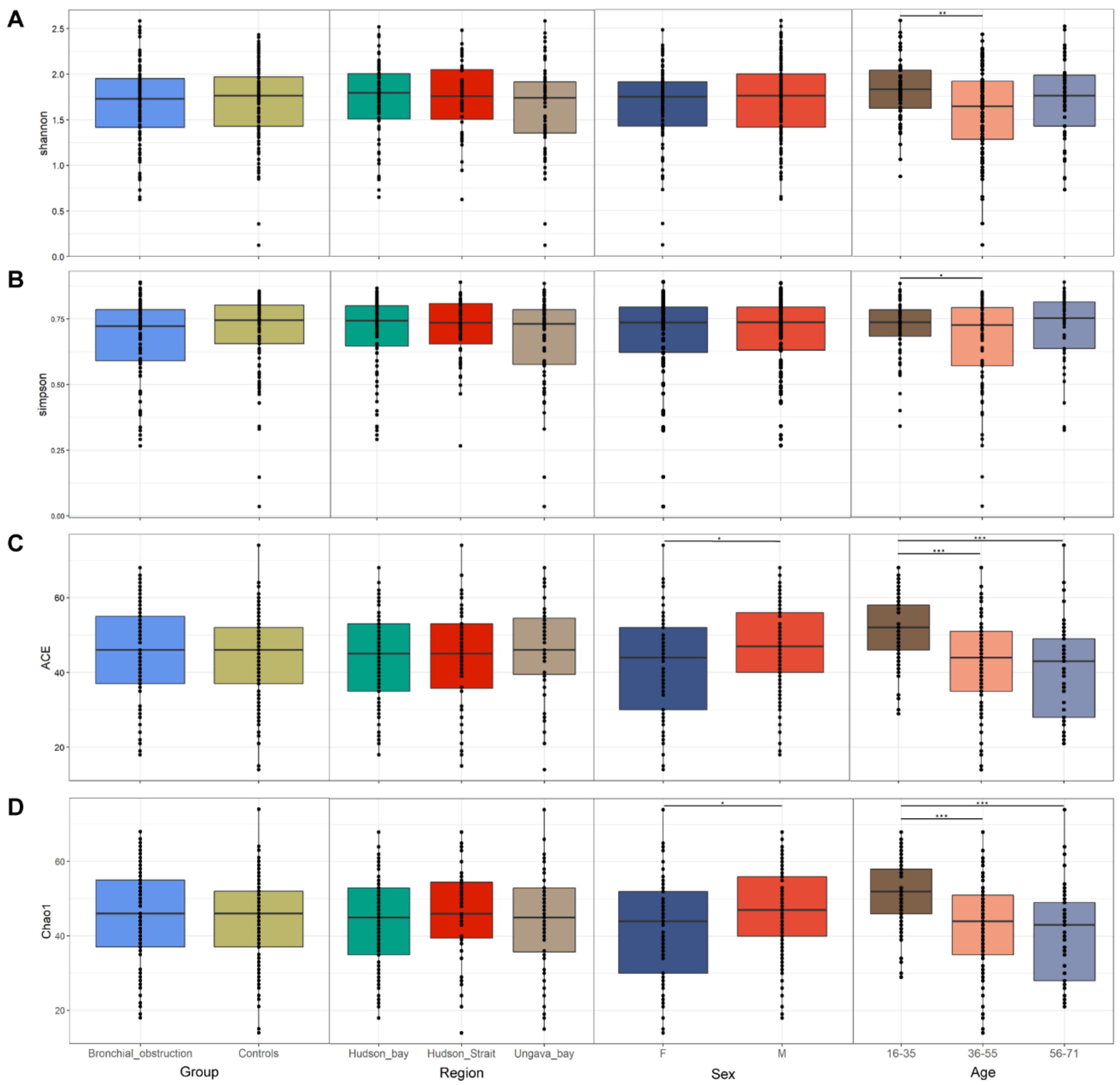

Supplementary figure 4. Extended α-diversity metrics of the oropharyngeal microbiome. α-diversity measured by Shannon index (A), Simpson index (B), ACE (C) and Chao1 (D) at the genus level. Each dot denotes the diversity of a sample. The diversities were measured based on the participants' respiratory capacities group ($FEV_1/FVC < 0.7$, bronchial obstruction group; $FEV_1/FVC > 0.7$, control group), their geographical origin (i.e. region), their sex, or their age. The boxes show inter-quartile ranges with the median denoted by a line. Significant differences are shown with Student's t-test for respiratory capacities group and sex data and analysis of variance (ANOVA) for geographical origin and age data. The Shannon index representation comparing the bronchial obstruction and control groups (i.e. top left plot) is the same as in Figure 4A.

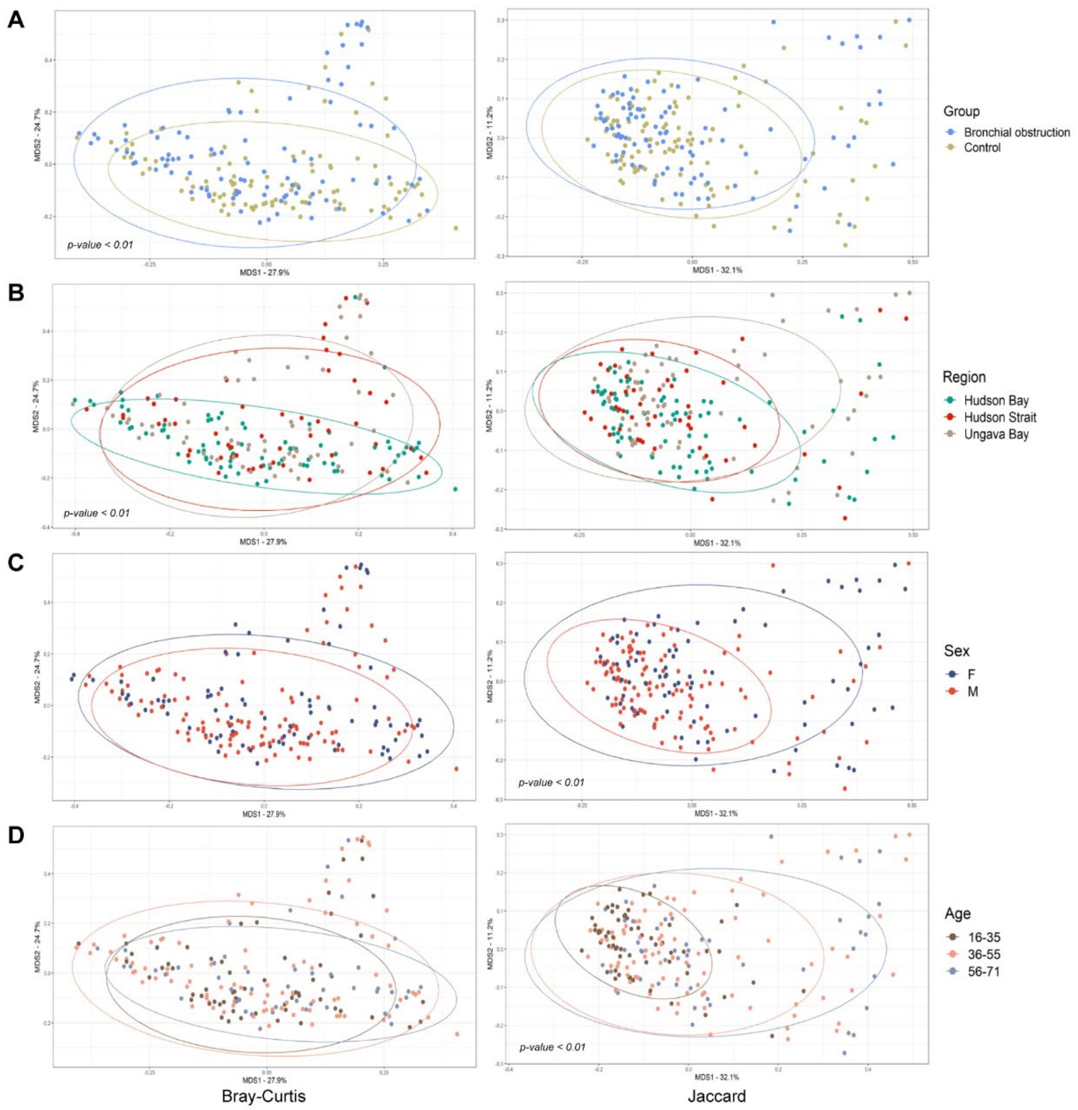

Supplementary figure 5. Extended β-diversity ordination of the oropharyngeal microbiome. β-diversity ordination was calculated at the genus level by non-metric multidimensional scaling (NMDS) using the Bray-Curtis dissimilarity (left) and Jaccard similarity index (right) of samples based on the participants' respiratory capacities group ($FEV_1/FVC < 0.7$, bronchial obstruction group; $FEV_1/FVC > 0.7$, control group) (A), their geographical origin (i.e. region) (B), their sex (C), or their age (D). The statistical significance of the clustering pattern in the ordination plot was evaluated using the Permutational ANOVA (PERMANOVA) and Analysis of group Similarities (ANOSIM) tests. The Bray-Curtis dissimilarity representation for the participants from the bronchial obstruction and control groups (i.e. top left plot) is the same as in Figure 4B.

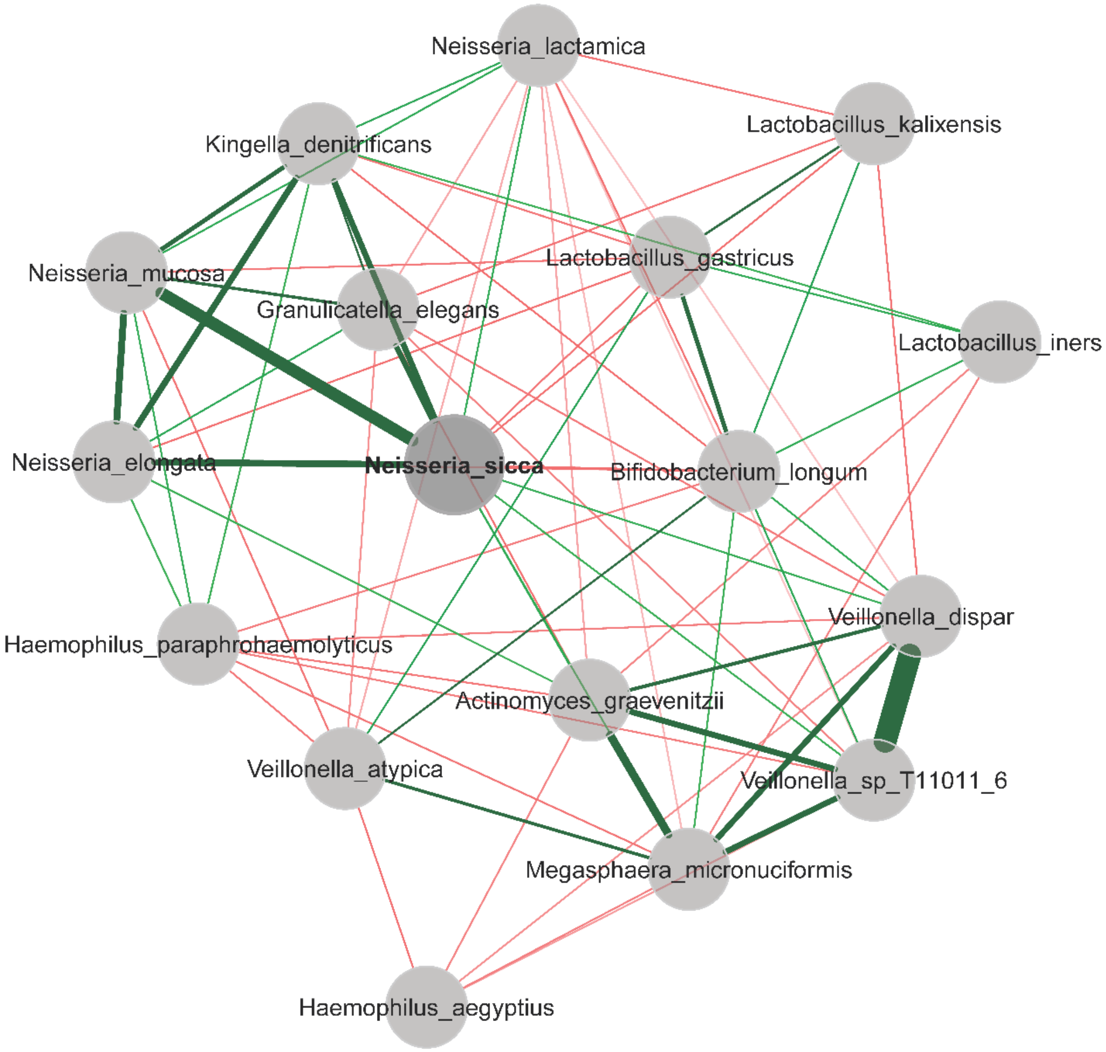

Supplementary figure 6. Sparse Correlations for Compositional data analysis for evaluating microbial co-occurrence among the bronchial obstruction and control groups. Nodes show the 17 species the most discriminant between the two groups according to the LEfSe analysis (see Figure 5A of the manuscript). Green lines denote species co-occurrence. Red lines denote the opposite. The width of the connector lines denotes the strength of the event.

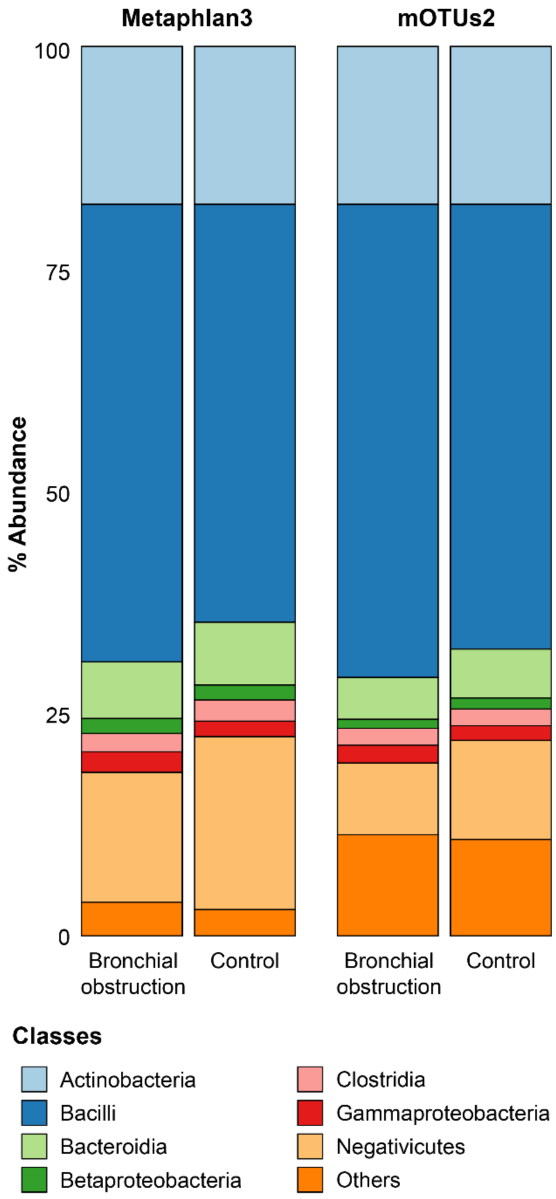

Supplementary figure 7. Relative abundance of bacterial classes in the two groups as obtained with MetaPhlAn3 and mOTUs2. Classes on the left are obtained with MetaPhlAn3 and classes on the right are obtained with mOTUs2. Only the bacterial classes with a relative abundance higher than 1% are depicted. These are the same whether MetaPhlAn3 (left) or mOTUs2 (right) was used for computing abundances. The abundances for the remaining bacterial classes were summed and shown by the 'Others' label. The barplot for Metaphlan3 is the same as in Figure 1.

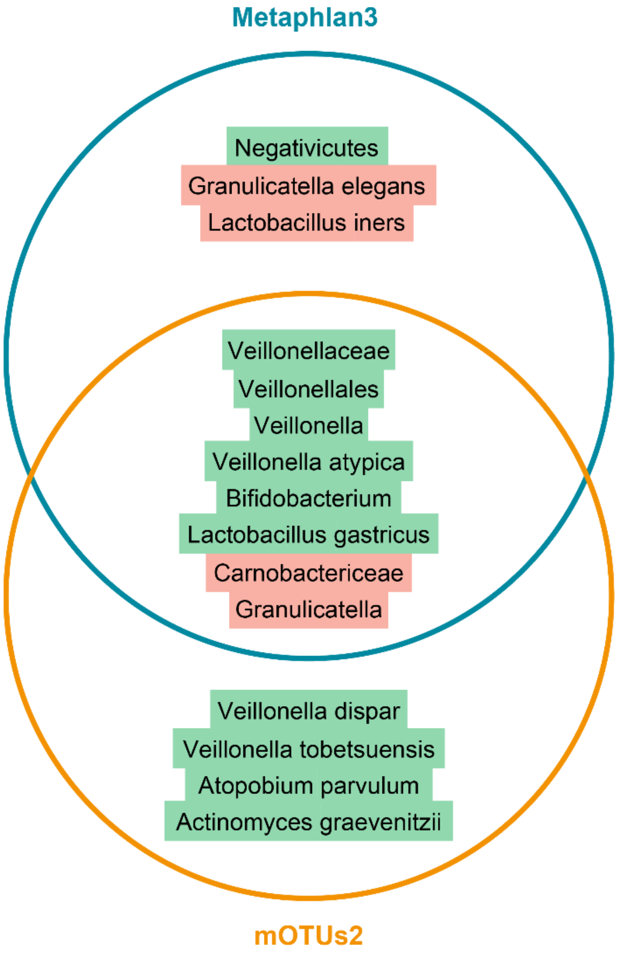

Supplementary figure 8. Venn diagram showing convergence of MetaPhlAn3 and mOTUs2 affiliations. The represented taxa are affiliated by MetaPhlAn3 (blue) and mOTUs2 (yellow) and emphasized by LEfSe analysis when comparing control (highlighted in green) against bronchial obstruction (highlighted in red) groups (LDA = 3, $p\text{-}value < 0.001$).

Supplementary table 1. Description of selected participants

		FEV1/VFC	
		**> 0.7**	**< 0.7**
**Sex**	Female	43	38
	Male	62	55
**Region**	Hudson Bay	41	38
	Ungava Bay	37	31
	Hudson Strait	27	24
**Age**	16-35	32	28
	36-55	47	42
	56 and +	26	23
**Smoking**	Non-smoker for life	9	10
	Former smoker	6	5
	Smoker	90	78
**Total**		105	93

The description is given in terms of sex, region, age, and smoking history with FEV1/FVC ratio obtained by spirometry during the Qanuilirpitaa? 2017 survey [see reference 6 in manuscript].

Supplementary table 2. Genera identified in the oropharyngeal microbiota by MetaPhlAn3 of Inuit participants.

Abiotrophia	Enorma	Parvimonas
Acetobacter	Enterococcus	Pasteurella
Acinetobacter	Escherichia	Patulibacter
Actinobaculum	Eubacterium	Peptoanaerobacter
Actinomyces	Facklamia	Peptococcus
Adhaeribacter	Faecalibacterium	Peptoniphilus
Aeriscardovia	Filifactor	Peptostreptococcus
Aerococcus	Finegoldia	Plantibacter
Aggregatibacter	Fretibacterium	Porphyromonas
Agrobacterium	Fusobacterium	Prevotella
Agrococcus	Gardnerella	Propionibacterium
Alistipes	Gemmata	Proteus
Alloiococcus	Granulicatella	Pseudoglutamicibacter
Alloprevotella	Haematobacter	Pseudomonas
Alloscardovia	Haemophilus	Pseudopropionibacterium
Anaerococcus	Janibacter	Pseudoramibacter
Anaeroglobus	Johnsonella	Psychrobacter
Anaerostipes	Kingella	Pyramidobacter
Aspergillus	Klebsiella	Ralstonia
Atopobium	Kocuria	Raoultella
Azospirillum	Kytococcus	Rhodococcus
Bacillus	Lachnoanaerobaculum	Roseburia
Bacteroides	Lactobacillus	Rothia
Barnesiella	Lactococcus	Ruminococcus
Bifidobacterium	Lautropia	Saccharomyces
Brevibacterium	Leptotrichia	Scardovia
Brevundimonas	Limnohabitans	Selenomonas
Bulleidia	Macrococcus	Serratia
Burkholderia	Mageeibacillus	Shuttleworthia
Campylobacter	Malassezia	Silicimonas
Candida	Megasphaera	Simonsiella
Candidatus Planktophila	Methanobrevibacter	Slackia
Capnocytophaga	Methylobacterium	Sneathia
Cardiobacterium	Methylorubrum	Solobacterium
Catonella	Microbacterium	Sphingomonas

Centipeda	Micrococcus	Staphylococcus
Chlamydia	Mitsuokella	Stenotrophomonas
Cloacibacterium	Mobiluncus	Stomatobaculum
Collinsella	Mogibacterium	Streptococcus
Corynebacterium	Moraxella	Tannerella
Criibacterium	Morococcus	Thermoleophilum
Cutibacterium	Mycoplasma	Thermus
Delftia	Neisseria	Treponema
Dermacoccus	Olsenella	Ureaplasma
Desulfobulbus	Oribacterium	Varibaculum
Desulfomicrobium	Ottowia	Variovorax
Dialister	Paludisphaera	Veillonella
Dolosigranulum	Pantoea	Xanthomonas
Eggerthia	Paracoccus	
Eikenella	Parascardovia	

Supplementary table 3. Description of publicly available datasets used for comparing metagenome populations.

		Datasets[a]			
		Castro-Nallar et al. 2015 [27]	de Castilhos et al. 2021 [29]	HMP	Retchless et al. 2020 [28]
**Samples' origin**		US	German	US	US
**# samples analysed (available)[b]**		8 (32)	239 (322)	14 (18)	66 (158)
**Age**		18-40	18-86	18-47	18-23
**Sex[c]**	**Female**	4	na	5	na
	**Male**	4	na	9	na
**Library preparation kit**		Nugen Ultralow DR Multiplex System	Nextera XT DNA Library Preparation	Illumina protocol	Nextera XT DNA library preparation
**DNA extraction kit**		Qiagen's Gentra Puregene Buccal Cell	QIAamp Fast DNA tissue	Mo Bio S3 PowerSoil DNA Isolation	QIAamp DNA Microbiome
**Sequencing platform**		Illumina HiSeq2000	Illumina NovaSeq 6000	Illumina GAIIx	Illumina HiSeq 2500 and MiSeq

[a] The numbers within brackets refer references in the manuscript. HMP, human microbiome project.

[b] The number of samples per external study analysed as part of the current study. The total number of samples available per external study is indicated within parentheses.

[c] na, not available.

Supplementary table 4. Hyperparameter tuning of the best feature selection method – machine learning algorithm combination for each microbiome data type.

Microbiome data	Feature selection method	Algorithm	Hyperparameter to tune	Hyperparameter optimal value	Accuracy score
MetaPhlAn, species level	Wilcoxon rank-sum test	Logistic regression	C	1	0.62
MetaPhlAn, genus level	Wilcoxon rank-sum test	Logistic regression	C	1	0.63
MetaPhlAn, family level	F-test, top 10%	Linear discriminant analysis	shrinkage	0.02	0.65
mOTUs, species level	Chi-squared test, top 30%	Decision tree	max_depth<br>min_samples_leaf	6<br>14	0.76
mOTUs, genus level	Chi-squared test, top 30%	AdaBoost	n_estimators<br>learning_rate	50<br>1	0.6
mOTUs, family level	F-test, top 10%	Linear discriminant analysis	shrinkage	None	0.62
HUMAnN	Logistic regression	AdaBoost	n_estimators<br>learning_rate	50<br>1	0.63
